# Stimulation of insulin secretion induced by low 4-cresol dose involves the RPS6KA3 signalling pathway

**DOI:** 10.1371/journal.pone.0310370

**Published:** 2024-10-24

**Authors:** François Brial, Géraldine Puel, Laurine Gonzalez, Jules Russick, Daniel Auld, Mark Lathrop, Roseline Poirier, Fumihiko Matsuda, Dominique Gauguier

**Affiliations:** 1 Université Paris Cité, INSERM U1132 Biologie de l’os et du cartilage (BIOSCAR), Paris, France; 2 Center for Genomic Medicine, Kyoto University Graduate School of Medicine, Kyoto, Japan; 3 Université Paris Cité, INSERM UMR 1124, Paris, France; 4 Institut des Neurosciences Paris-Saclay, Université Paris-Saclay, CNRS, Saclay, France; 5 Victor Philip Dahdaleh Institute of Genomic Medicine at McGill University, Montreal, QC, Canada; 6 Metabolica Drug Discovery Inc., Montreal, QC, Canada; Mae Fah Luang University School of Anti Aging and Regenerative Medicine, THAILAND

## Abstract

4-cresol (4-methylphenol, p-cresol) is a xenobiotic substance negatively correlated with type 2 diabetes and associated with health improvement in preclinical models of diabetes. We aimed at refining our understanding of the physiological role of this metabolite and identifying potential signalling mechanisms. Functional studies revealed that 4-cresol does not deteriorate insulin sensitivity in human primary adipocytes and exhibits an additive effect to that of insulin on insulin sensitivity in mouse C2C12 myoblasts. Experiments in mouse isolated islets showed that 4-cresol potentiates glucose induced insulin secretion. We demonstrated the absence of off target effects of 4-cresol on a panel of 44 pharmacological compounds. Screening large panels of 241 G protein-coupled receptors (GPCRs) and 468 kinases identified binding of 4-cresol only to TNK1, EIF2AK4 (GCN2) and RPS6KA3 (RSK2), a kinase strongly expressed in human and rat pancreatic islets. Islet expression of RPS6KA3 is reduced in spontaneously diabetic rats chronically treated with 4-cresol and *Rps6ka3* deficient mice exhibit reduction in both body weight and fasting glycemia, modest improvement in glycemic control and enhanced insulin release *in vivo*. Similar to low doses of 4-cresol, incubation of isolated rat islets with low concentrations of the RPS6KA3 inhibitor BIX 02565 stimulates both glucose induced insulin secretion and β-cell proliferation. These results provide further information on the role of low 4-cresol doses in the regulation of insulin secretion.

## Introduction

Multifactorial disorders result from the combined effects of many genes, lifestyle and environmental factors. Genome wide association studies have revealed many disease risk loci, each explaining a modest proportion of the disease risk [1,2]. Growing evidence supports a role of environmental pollutants in the aetiology of chronic diseases. For example, recent epidemiological studies have demonstrated that the consumption of ultraprocessed food containing additives, contact materials and neoformed contaminants increases the risk of cardiovascular diseases [3] and cancer [4]. Dietary artificial additives and endocrine disruptors increase the risk of metabolic diseases [5–7]. The microbiota is also an important component in environmental exposures that contributes to the risk of multifactorial diseases through its impact on host metabolic regulations and the maturation of the immune system [8,9].

4-cresol (p-cresol, 4-methylphenol) is a substance naturally present in food, drinks, cigarette smoke, wood burning and surface waters and groundwater (www.atsdr.cdc.gov/), which can be absorbed by ingestion, inhalation or dermal contact. 4-cresol is also synthesised by colonic fermentation of tyrosine and phenylalanine by bacteria of the gut microbiota [10] via decarboxylation of para-hydroxyphenylacetate by the decarboxylase encoded by the operon *hpdBCA* [11], as well as presumably by protein degradation. Lethal dose (LD50) for 4-cresol ranges from 200 to 5000 mg/kg/day in rodents [12]. Dietary exposure to high doses of 4-cresol (240-2000mg/kg/day) causes irritation to the respiratory epithelium and liver dysfunction [13].

We have recently demonstrated a negative correlation between plasma 4-cresol and type 2 diabetes in humans [14]. Subsequent studies in preclinical models of experimentally-induced and spontaneously-occurring diabetes showed that chronic infusion of low doses of 4-cresol (0.5 mg/kg/day) reduces adiposity and fatty liver, improves glucose tolerance and enhances insulin secretion, islet neogenesis and β-cell proliferation [14].

4-cresol may have much broader biological roles. Mouse studies suggested that exposure to 4-cresol induces autistic-like phenotypes [15] and reduces intestinal transit and the transcription of gut hormones [16], even though the administration of this volatile compound in the drinking water and its known toxic effects on respiratory mucosae undermine the conclusions of these reports. We recently showed that plasma levels of its detoxification product (4-cresyl sulfate) are associated with multiple endophenotpyes in humans suggesting its pleiotropic biological roles [17]. Urine levels of 4-cresyl sulfate are also negatively associated with body mass index in humans [18]. Inhibition of proliferation and differentiation of 3T3-L1 preadipocytes by 4-cresol supports results from these association studies [19,20].

To further evaluate the physiological roles of low doses of 4-cresol, we carried out a series of investigations to rule out its off target effects on pharmacological compounds, to test its impact on insulin sensitivity and secretion *in vitro*, and to identify molecular targets followed by experimental validation in preclinical models. Results from our study allow revision of current estimates of the risk of exposure to 4-cresol and provide further information on its role in the regulation of insulin secretion.

## Material and methods

### Animals

Adult male C57BL/6J mice and Wistar rats (6–7 week-old) purchased from a commercial supplier (Janvier Labs, Le Genest St Isle, France) were used for islet isolation. Twenty week-old male *Rps6ka*3^-/-^mice [21] and wild-type male littermates were bred and maintained in the laboratory. Animals were housed in conventional animal care unit. They were fed a standard chow diet (R04-40, Safe, Augy, France) ad libitum and were maintained under controlled conditions of temperature (22 ± 3°C), humidity (50 ± 20%) and photoperiod (12h light/12h dark). All procedures were authorized following review by the institutional ethic committee and were carried out under national licence condition.

Animals were housed in cages enriched with nesting material and toys. An intraperitoneal injection of ketamine (100 mg/kg) and xylazine (10 mg/kg) was administered to induce deep anaesthesia. To reduce animal suffering, animals were frequently monitored for signs of pain. Animals were sacrificed by cervical dislocation.

All animal procedures described in this study were approved by the Charles Darwin Animal Experimentation Ethics Committee, the institutional ethics committee of CEEA-005, and were performed in accordance with the French licensing conditions (ref. 00486.02). The authors followed the ARRIVE guidelines.

### Glucose tolerance and insulin secretion tests

Groups of six *Rps6ka3*^-/-^ and wild-type mice were fasted overnight prior to the intraperitoneal glucose tolerance test (IPGTT). A blood sample was collected through the tail vein to acclimatize the animals to blood sampling. Thirty minutes later, a solution of D-Glucose (2 g/kg) was injected intraperitoneally in conscious animals. Blood was collected from the tail vein before glucose injection and 15, 30, 60 and 120 minutes afterwards. Blood glucose levels were immediately determined using an Accu-Check® Performa (Roche Diagnostics, Meylan, France). Additional blood samples were collected at baseline and 15 and 120 minutes after glucose injection in Microvette® CB 300 Lithium Heparin (Sarstedt, Marnay, France) for subsequent insulin assay. Plasma was separated by centrifugation and stored at -80°C until insulin assays using ELISA kits (Mercodia, Uppsala, Sweden). Evaluation of overall glucose tolerance was obtained from the cumulative glycemia, which was determined by the total increment of plasma glucose values during the IPGTT (Area Under the Curve, AUC).

### Pancreas immunohistochemistry

Pancreas sections (5 μm) of Goto-Kakizaki rats chronically treated with subcutaneous delivery of either 4-cresol or saline as part of a previous study [14] were used. They were quenched with 3% H2O2, washed with TBS + 0.1% (v/v) Tween-20 (or 0.05% v/v Triton X-100 for nuclear epitopes), blocked with TBS + 3% (w/v) BSA, and incubated with a solution of diluted primary antibody anti-*Rps6ka3* (ab111068, Abcam, Paris, France) and then with PolyStain 2-Step Plus Kit, HRP, Rabbit, with DAB (Clinisciences, Nanterre, France). Nuclei were counterstained with hematoxylin. Quantitative analysis of all immunostainings was performed using an open source software (https://qupath.github.io).

### Glucose uptake in mouse C2C12 myotubes

Mouse C2C12 myoblast cells were purchased from ATCC and maintained in a growth medium (Dulbecco’s Modified Eagle Media (DMEM) 4.5g/L glucose supplemented with 10% fetal bovine serum, 100U/ml penicillin, and 100μg/ml streptomycin) at 37°C in a humidified atmosphere of 95% air / 5% CO2. For the differentiation step, cells were grown in 96-well-plates at a density of 10,000 cells/well in 0.2mL of growth medium. 24 hours after plating, the differentiation induction into multinucleated myotubes was carried out in DMEM 4.5g/L glucose containing 2% fetal bovine serum. After 5 days, glucose uptake was determined. Cells were washed with Krebs buffer and incubated with 4-cresol (10 and 30 nM) in the absence or in the presence of submaximal concentrations of insulin (10^−8^ M and 2.10^−8^ M) for 4 hours in DMEM containing 1g/L glucose, 0.25% BSA, 100U/ml penicillin, and 100μg/ml streptomycin (n = 6 per condition). Insulin (10^-6^M) was used as reference. At the end of treatment, glucose uptake was measured by incubation of the cells with 2-deoxy-D-Glucose (0.5 mM), which was phosphorylated to form 2-deoxy-D-Glucose-6-Phosphate (2DG6P) that accumulates into the cells. 2DG6P was estimated using the bioluminescent Glucose-GloAssay (Promega, Charbonnières-les-Bains, France).

### Glucose uptake in human adipocytes

Cryopreserved human subcutaneous preadipocytes used in the study were obtained for a commercial supplier (Zen-Bio, Durham, NC). The donor is male gender, 49-year-old, with a BMI of 24.5 kg/m^2^. Cryopreserved human subcutaneous preadipocytes were thawed and plated onto 96-well plates at density of 15,000 cells/well. Preadipocytes were differentiated over a period of 14 days until incubation in a medium provided by the commercial supplier of the human preadipocytes (Zen-Bio, Durham, NC, US) depleted in insulin, serum and glucose. Cells were treated for 4 hours with 4-cresol (10 and 30 nM) and insulin (1.0x10^-8^ M and 1.0x10^-9^ M) (n = 8 per condition) until addition of 2-deoxy-D-Glucose (1 mM) for 10 min to measure glucose uptake using the bioluminescent Glucose-GloTM assay (J1342, Promega, Charbonnières-les-Bains, France). 2-deoxy-D-Glucose phosphorylation was quantified using the bioluminescent Glucose-GloTMAssay (Promega, Charbonnières-les-Bains, France). Protein quantification was determined using a colorimetric assay (PIERCE 22663 & 22660, Life Technologies, Saint-Aubin, France).

### Mouse islet isolation and *in vitro* insulin secretion analysis

Pools of islets from six mice were isolated by collagenase digestion of the pancreas and washed in Hanks balanced salt solution (HBSS) (Sigma Aldrich, St Quentin, France) followed by purification using Histopaque 1077 (Sigma Aldrich, St Quentin, France) and HBSS. Islets were handpicking at a density of 50 islets into Petri dishes and placed in RPMI 1640 (Sigma Aldrich, St Quentin, France) supplemented with 10 mM Hepes, 2mM glutamine, 100 U/ml Penicillin, 100 μg/ml Streptomycin and 10% calf serum at 37°C. Following a 72 hours stabilization period, islets were pre-incubated for 30 min in Krebs-BSA 0.2% with 2.8 mM glucose. To test the acute effects of 4-cresol on insulin secretion, islets were dispatched in 6 islets per well for 60 minutes in Krebs BSA 0.2% with 2.8 mM, 8 mM or 16.7 mM of glucose in the absence (controls) or the presence of 4-cresol (5 nM, 10 nM, 20 nM and 40 nM). Incubation with the glucagon-like peptide-1 GLP-1 (100 nM) (Sigma Aldrich, St Quentin, France) was used as reference in the presence of glucose 16.7 mM. In addition, a chronic 48 hours-treatment of islets with 4-cresol was tested in parallel. At the end of the 90 minutes incubation period, supernatants were removed and stored at -20°C until ELISA assay of insulin (Alpco, Salem, US).

### Fat accumulation in 3T3-L1 cells treated with RPS6KA3 inhibitors

3T3-L1 mouse preadipocytes were a gift from METABRAIN SA. Cells were cultured in 5% CO2 at 37°C in humid conditions in high-glucose Dulbecco’s modified Eagle’s medium (DMEM) (Sigma Aldrich, St Quentin, France), supplemented with 10% fetal bovine serum (FBS) and 1% penicillin–streptomycin (ThermoFisher Scientific, Villebon,France). The medium was changed twice a week. For induction of differentiation, after reaching confluency the cells were cultured in DMEM supplemented with 10% FBS, 1% penicillin–streptomycin, 1 μg/mL insulin, 0.25 μM dexamethasone, and 0.5 mM 3-isobutyl-lmethylxanthine. Differentiated cells were incubated with RPS6KA3 inhibitors FMK and BIX 02565 (2 μM and 10 μM). Cells were then labeled with BODIPY 493/503 (boron-dipyrromethene) (Invitrogen, ThermoFisher Scientific, Villebon, France), a marker for fat accumulation in live cells. Briefly, after treatment with inhibitors, the cells were washed twice and incubated in emulsified 1X Bodipy solution for 30 minutes at 37°C. The Mean Fluorescence Intensity corresponding to Bodipy was acquired on Attune flow cytometer (ThermoFischer Scientific, Villebon, France), in the FITC channel. Analysis was performed using the FlowJo software (www.flowjo.com).

### Effects of RPS6KA3 inhibitors on *in vitro* insulin secretion and β-cell proliferation

Six-week-old Wistar rats were anesthetized and the pancreas was dissected. The pancreas was digested in collagenase for 10 minutes. The reaction was stopped by adding HBSS (Hank’s Balanced Salt solution) buffer (Sigma Aldrich, St Quentin, France). After centrifugation, the supernatant was removed and the islets were pooled and resuspended in Hanks buffer. This process was repeated three times and the islets were stabilized for 72 hours in RPMI 1640 (Sigma Aldrich, St Quentin, France). Then, islets were pre-incubated for 30 minutes in Krebs-BSA 0.2% with 2.8 mM glucose.

For the insulin secretion experiments, islets were aliquoted in 6 islets per well in 24 well plates and incubated for 48 hours with 4-cresol 10 nM or the RPS6KA3 inhibitors FMK and BIX 02565 at concentrations of 2 μM and 10 μM, which are within the range of previously reported experiments in pancreatic islets [22]. Insulin secretion was tested in response to static incubation of the islet preparations with glucose 2.8, 8.0 or 16.7 mM for 90 minutes. Incubation of islets with GLP-1 100 nM (Sigma Aldrich, St Quentin, France) in the presence of glucose 16.7mM was used to assess the quality of the islet preparations. At the end of the 90 minutes incubation period, supernatants were removed and stored at -20°C until ELISA assay of insulin (Alpco, Salem, US).

To analyse cell proliferation in isolated islets, a separate batch of islets was picked manually at a density 50 islets in Petri dishes and maintained overnight at 37°C in a solution containing RPMI1640 and Hepes (10 mM), glutamine (2 mM), Penicillin (100 U/ml), Streptomycin (100 μg/ml) and 10% SVF. Islets were then incubated for 48 hours in a medium containing either each of the two kinase inhibitors FMK and BIX 02565 at 2 μM or GLP1 (10 nM) (Sigma Aldrich, St Quentin, France). The two inhibitors and GLP-1 were prepared in H2O and diluted in RPMI 1640 completed with 10% SVF to achieve the final tested concentration. Media were changed every 24h. Then 40 islets were collected for each condition, washed in Dulbecco’s phosphate-buffered saline (DPBS, Sigma Aldrich, St Quentin, France) and digested by Trypsin-EDTA 0.25%. Cytospin was performed with islets fixed in paraformaldehyde 3.7% and permeabilized with Triton X100–0.2% in BSA 5%. Islet preparations were stained with a rabbit anti-Ki67 antibody (Abcam, Paris, France) coupled with goat anti-rabbit IgG Alexa Fluor 594 (ThermoFisher Scientific, Villebon, France). β-cell nuclei were stained with a polyclonal guinea pig anti-insulin antibody (Agilent DAKO, Courtaboeuf, France) coupled with an anti-guinea pig antibody conjugated with FITC. Hoechst 33342 (ref. H3570) (ThermoFisherScientific, Villebon, France). The number of cells stained with Ki67 or insulin immunostaining was determined after slide scanning and analysis with the CaseViewer Software (www.3dhistech.com/solutions/caseviewer/). Scanning was performed on 3 to 4 slides per group. Over 6,000 cells per group in randomly selected fields were counted to determine the total number of nuclei counted, the percentage of beta cells, the percentage of proliferating cells and the percentage of proliferating β-cells.

### Analysis of off-target effects of 4-cresol

4-cresol (1.0x10^-5^ M) was tested in duplicate for its capacity to bind *in vitro* to 44 compounds selected for their pharmacological importance in the commercial panel SafetyScreen44™ (Eurofins Cerep, Le Bois l’Evêque, France), or affect their function, and therefore to verify that 4-cresol is not an unsafe compound. The concentration of 4-cresol used for the assay (1.0x10^-5^ M) followed the Eurofins’ recommendation. Experiments were carried out according to the supplier’s protocol. The binding or functional effects of 4-cresol were calculated as the percentage of inhibition of the binding of a radioactively labeled ligand specific to the target or the percentage of inhibition of control enzyme activity. For each experiment, a reference compound was tested concurrently with 4-cresol, and the data were compared with historical values determined at Eurofins. The experiment was analysed according to Eurofins validation Standard Operating Procedure. Changes in binding or inhibition values above 50% were considered as significant, whereas variations in the range of 25–50% and below 25% were considered as weak to moderate and not significant, respectively.

### Analysis G protein-coupled receptors (GPCRs) activation by 4-cresol

PathHunter assays based on the enzyme fragment complementation (EFC) technology developed by DiscoverX was used to analyse potential effects of 4-cresol on activation or internalization of 168 known GPCRs using the gpcrMAX panel and 73 orphan GPCRs using the orphanMax panel (Eurofins DiscoverX, Le Bois l’Evêque, France). These analyses were carried out with a single concentration of 4-cresol (10 μM). As above, the concentration of 4-cresol used for the assay followed the supplier’s recommendation. All experiments of agonist and antagonist determination were carried out in cell lines according to protocols of the commercial supplier.

### Kinase assay and binding affinity prediction of 4-cresol

To identify candidate protein targets of 4-cresol, we used the scanMax of the KINOMEscan™ Profiling Service (Eurofins Cerep, Le Bois l’Evêque, France). It is an active site-directed competition binding assay to quantitatively measure interactions between test compounds and 468 human kinases. 4-cresol was tested at 10μM according to Eurofins recommendation. Binding interactions of 4-cresol to the kinases were evaluated with respect to values obtained with the positive control (the actual compound) and the negative control (DMSO). Selectivity scores were calculated as the ratio of the number of kinases that 4-cresol binds to and the total number of distinct kinases tested. A threshold of 65% binding inhibition was used to assess significant interaction as recommended by the commercial supplier.

### Statistical analysis

To assess the significance of the effects of 4-cresol on glucose tolerance, insulin secretion *in vivo* an *in vitro*, and glucose uptake *in vitro*, statistical analyses were performed using an Anova followed by a Dunnett’s test or a Kruskal-Wallis followed by a Dunn’s test if variances significantly differ. A p value of < 0.05 was considered as significant.

## Results

### 4-cresol exhibits an additive effect to that of insulin on glucose uptake in mouse C2C12 myotubes

**Fig 1:**
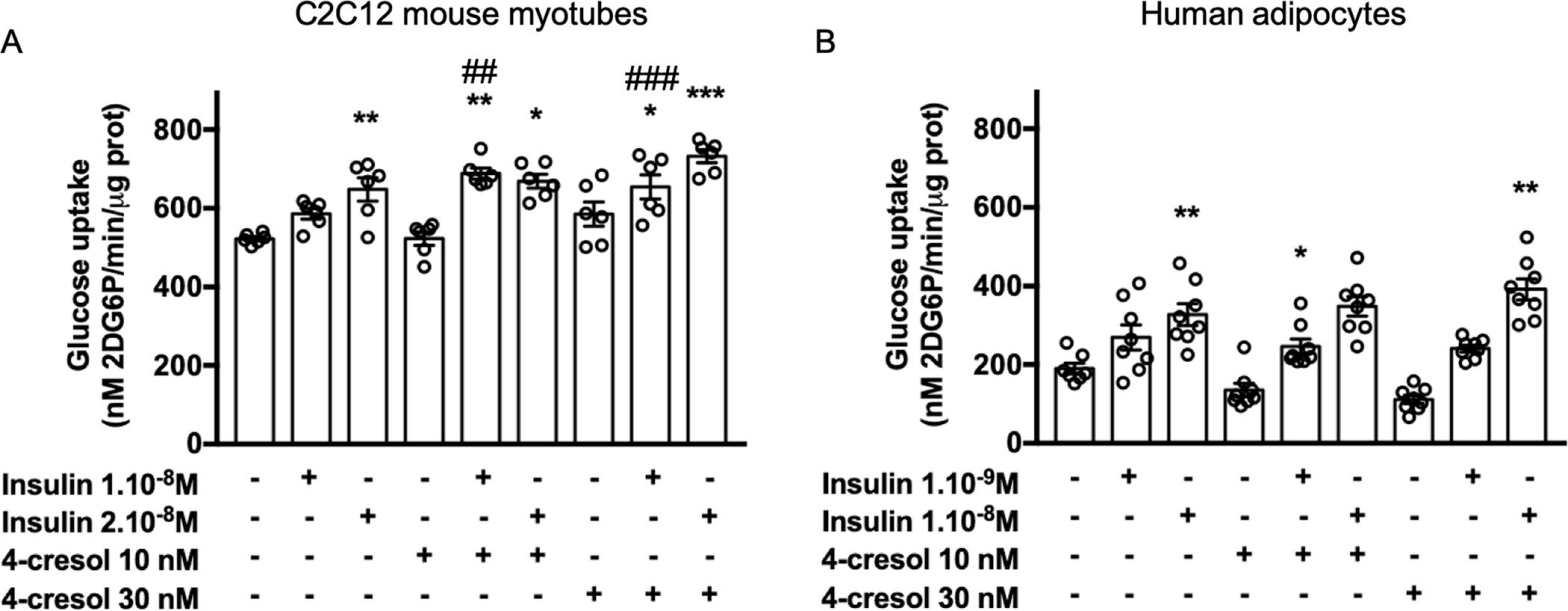
Effect of insulin and 4-cresol on glucose uptake in C2C12 mouse myotubes (A) and in human adipocytes (B). Glucose uptake was evaluated by measuring the phosphorylation of 2-deoxy-D-Glucose (2DG6P) after a 4h-incubation with vehicle (control) or 4-cresol (10 or 30 nM) in combination with insulin 1.0x10^-8^ M and 2.10x10^-8^M in C2C12 (A) and with insulin 10^-9^M and 10^-8^M in human adipocytes (B). Results were expressed in nM/min/μg of proteins. Statistical analyses were performed using a Kruskal-Wallis followed by a Dunn’s multiple comparison test. Results are means ± SEM. *p<0.05, **p<0.01, ***p<0.001 significantly different to data in the absence of 4-cresol and insulin. ^##^p<0.01, ^###^p<0.001 significantly different to the sum of effects of corresponding concentrations of 4-cresol and insulin on glucose uptake. n = 6 observations per treatment in C2C12 and n = 8 observations per treatment in human adipocytes.

To test the effects of 4-cresol on insulin sensitivity *in vitro*, we initially analysed glucose uptake in the C2C12 myotube model of mouse myocytes. As expected, a dose-dependent increase in glucose uptake by C2C12 myotubes was induced by a 4h exposure to insulin 1.0x10^-8^ M (+12%) and 2.0x10^-8^M (+24%, p<0.05) (**Fig 1A**). Glucose uptake was not significantly altered in C2C12 myotubes incubated for 4h with 4-cresol alone at 10 nM (0% change) and 30nM (+12%). Exposure to combinations of 4-cresol (10nM and 30nM) and insulin (1.0x10^-8^ M and 2.0x10^-8^ M) significantly increased glucose uptake when compared to controls incubated in the absence of insulin and 4-cresol (**Fig 1A**). A synergistic effect of 4-cresol (10 nM) and insulin (1.0x10^-8^ M) on glucose uptake was observed as their combined effect was significantly greater (+32%, p<0.001) than the sum of the individual effects of 4-cresol (0%) or insulin (+12%). We showed that incubation of C2C12 cells with an increased dose of 4-cresol (30nM) also potentiated the effects of insulin (1.0x10^-8^ M) on glucose uptake (+25%, p<0.01 vs control) to a level corresponding to the sum of the individual effects of 4-cresol (+12%) and insulin (+12%) (**Fig 1A**). These results demonstrate the additive effect of 4-cresol to that of insulin on glucose uptake in C2C12 cells.

### 4-cresol does not affect glucose uptake in human adipocytes

To test whether results from glucose uptake obtained in a mouse model of myocytes can be translated in human cells, we replicated the same experiment in human primary adipocytes. As expected, adipocytes exhibited a dose-dependent stimulation of glucose uptake when incubated with the increasing doses of insulin 1.0x10^-9^ M (+42%) and 1.0x10^-8^ M (+72%, p<0.01) (**Fig 1B**). In non-physiological conditions (ie. absence of insulin), a 4h-exposure of human adipocytes to 4-cresol decreased glucose uptake when compared to control conditions (-29% at 4-cresol 10 nM; -42% at 4-cresol 30 nM). In contrast, glucose uptake was consistently increased when adipocytes were incubated with insulin 10^-9^M and 4-cresol 10 nM (+27%) and 30 nM (+29%) but differences to controls were statistically significant only at 4-cresol 10 nM (**Fig 1B**). These effects of 4-cresol on stimulated glucose uptake were nevertheless significant when compared to conditions where adipocytes were treated with 4-cresol in the absence of insulin (+78% at 4-cresol 10 nM, p<0.05 and +122% at 4-cresol 30 nM, p<0.01). Incubation of adipocytes with 4-cresol at 10 nM or 30 nM did not result in additive or potentiating effect of insulin 1.0x10^-9^ M on glucose uptake (**Fig 1B**). The combined effects of a 4h-exposure of 4-cresol (10 nM or 30 nM) and insulin on the stimulation of glucose uptake were amplified (+83% to +106%, p<0.05) when adipocytes were incubated with an increased concentration of insulin (1.0x10^-8^ M), but 4-cresol still showed little additive impact (up to 20%) to that of insulin on glucose uptake (**Fig 1B**).

### 4-cresol stimulates insulin secretion *in vitro* in isolated mouse islets

**Fig 2:**
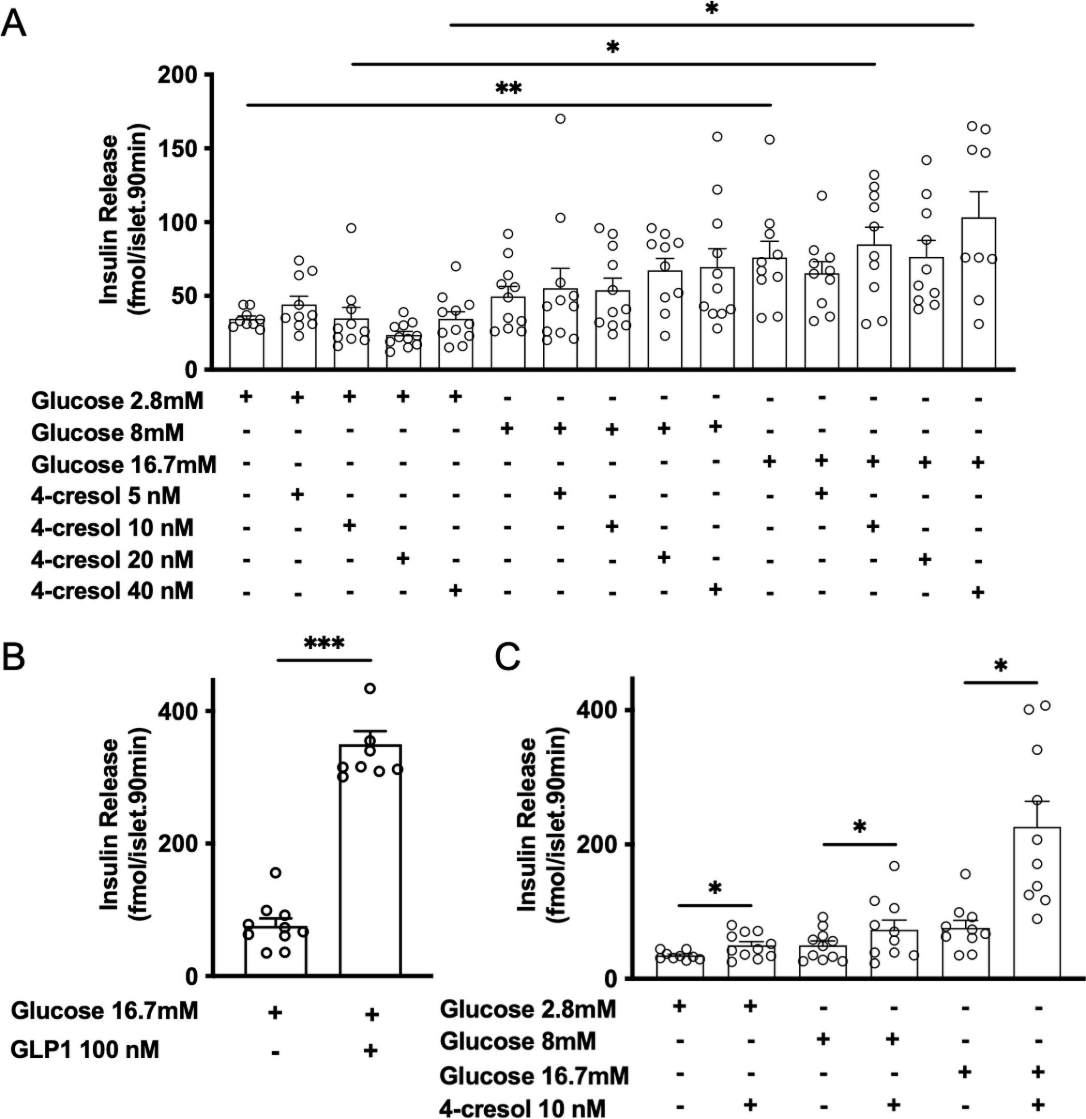
Effect of 4-cresol on glucose-induced insulin in isolated mouse islets. Islets prepared from C57Bl/6J mice were incubated 90 minutes with glucose (2.8 mM, 8 mM and 16.7 mM) in the absence (control) or in combination with 4-cresol (5 nM, 10 nM, 20 nM, 40 nM) (A) and the glucagon-like peptide-1 (GLP-1) (100 nM) (B). In a separate experiment, islets were chronically exposed to 4-cresol 10 nM or vehicle for 48 hours prior to incubation with glucose (2.8 mM, 8 mM and 16.7 mM) for 90 minutes (C). Insulin levels were measured in supernatants after 90 minutes of incubation using an ELISA test. Each point represents mean values of six islets. Statistical analyses were performed using an Anova followed by a Dunnett’s test. Results are means ± SEM. *p<0.05, **p<0.01, ***p<0.001 significantly different to insulin values in the absence of GLP-1 in (B) and 4-cresol in (C). N = 9 to 12 islet pools per group.

We previously showed that 4-cresol at a concentration below 100 nM stimulates *in vitro* glucose induced insulin secretion in mouse isolated islets [14]. To refine the minimal concentrations of 4-cresol able to stimulate insulin secretion, we repeated the experiment in mouse isolated islets incubated with 4-cresol at 5 nM, 10 nM, 20 nM and 40 nM. After 90 minutes of incubation of mouse islets with glucose, we observed a dose-dependent increase in insulin response to glucose 8 mM (+44%) and 16.7 mM glucose (+120%, p<0.01) when compared to the response to 2.8 mM glucose (**Fig 2A**). GLP-1 (100 nM) significantly increased insulin release in response to 16.7 mM glucose (+361%, p<0.001) (**Fig 2B**). Insulin secretion in response to glucose 8 mM was increased by up to 186% when islets was incubated with 4-cresol 20 nM. In the presence of glucose 2.8 mM, 8 mM or 16.7 mM, 4-cresol did not significantly potentiate insulin release at four concentrations tested (5, 10, 20, 40 nM) (**Fig 2A**). In contrast, prior prolonged 48 hours-treatment of islets with 4-cresol 10 nM resulted in a strong enhancement of insulin release in response to glucose 2.8 mM (+44%, p<0.05) and insulin secretion stimulated by glucose 8 mM (+48%, p<0.05) and 16.7 mM (+200%, p<0.01) (**Fig 2C**). These data suggest that chronic exposure of islets to 4-cresol is required to stimulate insulin secretion *in vitro*.

### 4-cresol does not significantly bind pharmacological targets or affect their function

**Fig 3:**
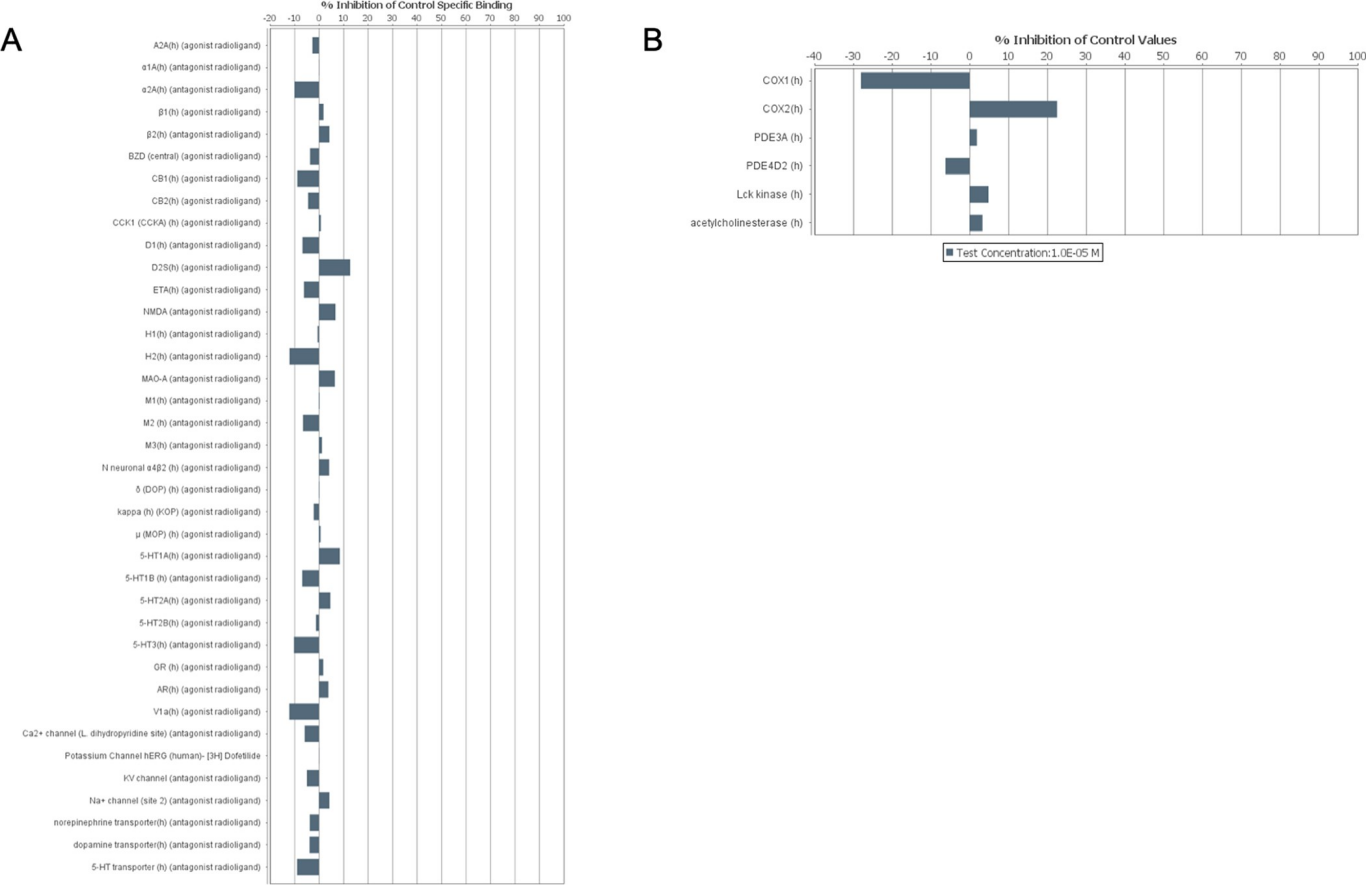
Analysis of potential off target effects of 4-cresol. The Eurofins Safety-Screen 44 panel was used for *in vitro* pharmacological profiling and assessment of off-target interactions of 4-cresol with leading compounds in binding screens. 4-cresol was tested in duplicate at a concentration of 10 μM for its potential to interfere with the binding of native ligands of 44 receptors, transporters, ion channels and enzymes.

To evaluate the safety of 4-cresol through the identification of possible off-target interactions with compounds of pharmacological importance, we tested 4-cresol (1.0x10^-5^ M) on a commercial assay against a panel of 44 pharmaceutical compounds (GPCRs, transporters, ion channels, nuclear receptors, kinases and non-kinase enzymes). Based on the recommended cut-off value of 50% binding inhibition (or stimulation for assays run in basal conditions), we did not find evidence of significant binding or functional interference of 4-cresol to any pharmacological targets (**Fig 3A and 3B**). The strongest evidence of altered binding induced by 4-cresol was observed for COX1 (-27%) and COX2 (+22%). The remaining targets showed binding values in the range of ±12%. These results suggest that non-toxic effects of low doses of 4-cresol are specific and do not affect pharmacologically important targets, portending favourable therapeutic and safety profiles.

### Screening 4-cresol interaction with G protein-coupled receptors (GPCRs)

In order to understand the mechanism of action of 4-cresol at the cellular level, we undertook a systematic *in vitro* functional screening of GPCRs using the PathHunter β‐arrestin enzyme fragment complementation (EFC) technology allowing analysis of the effects of 4-cresol on 168 known and 73 orphan GPCRs (**S1 Table**). According to effect threshold criteria set by the commercial supplier (% activity >30% in agonist mode and % inhibition >35% in antagonist mode for the gpcrMax panel; % activity >50% for the orphanMax panel), these assays failed to reveal significant interactions between 4-cresol and the GPCRs. Nevertheless, at the concentration of 4-cresol tested (10 μM), we identified relevant effects of this metabolite (over ±25% of effects) on the inhibition of prolactin releasing hormone receptor (PRLHR) (-27%), the somatostatin receptor 5(SSTR5) (-25%), GPR119 (+26%) and the opioid receptor-like 1(OPRL1) (+27%) and the activity of the orphan GPCRs GPR27 (+27%) and GPR132 (+33%) (**S1 Table)**.

### 4-cresol binds three kinases

**Fig 4:**
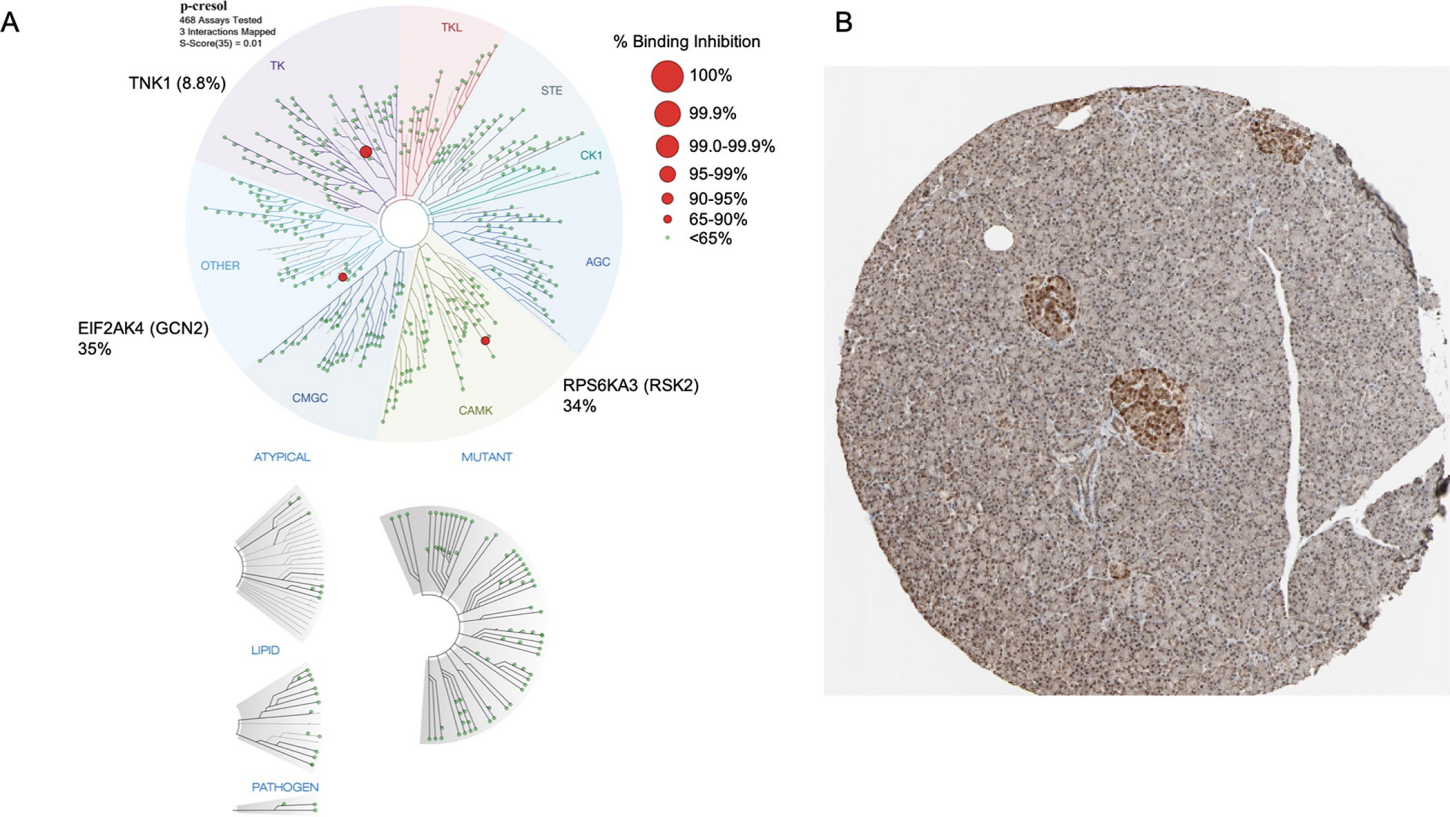
RPS6KA3 binds 4-cresol and is a candidate mediator of its effects on diabetes. Interaction maps for 4-cresol against the panel of kinases are shown in a representation of the human kinome phylogenetic tree (A). Kinases that bind to 4-cresol are marked with red circles, where larger circles indicate higher-affinity binding. The full list of 468 kinases tested in the interaction assays and the percentage of binding inhibition to the proteins by 4-cresol are given in **S2 Table**. Evidence of RPS6KA3 expression in human pancreatic islets was assessed by *in situ* hybridization (source: The Human Protein Atlas, www.proteinatlas.org/search/RPS6KA3) (B).

To identify candidate proteins mediating the impact of 4-cresol on metabolism of glucose and the secretion of insulin and investigate underlying molecular mechanisms, we screened an extensive panel of 468 kinases for possible interference with 4-cresol (**S2 Table**). 4-cresol showed no evidence of significant interference with 214 kinases (100% binding to kinase remaining after treatment with 4-cresol) and at least 25% of binding inhibition for 23 kinases. We showed that 4-cresol strongly interacts with the tyrosine kinase non receptor 1 (TNK1) (91.2% binding inhibition), the ribosomal protein S6 kinase A3 (RPS6KA3, RSK2) (66% binding inhibition) and the eukaryotic translation initiation factor 2 alpha kinase 4 (EIF2AK4) (65% binding inhibition) (**Fig 4A and S2 Tables**). Among these, mining available data in the human proteome atlas (www.proteinatlas.org) showed that RPS6KA3 is highly expressed in pancreatic islets (**Fig 4B**) and is therefore a strong candidate to mediate the role of 4-cresol on the stimulation of insulin secretion that we demonstrate here *in vitro* and on the previously reported enhancement of β-cell proliferation [14].

### The kinase RPS6KA3 is a candidate to mediate the effects of 4-cresol on diabetes

**Fig 5:**
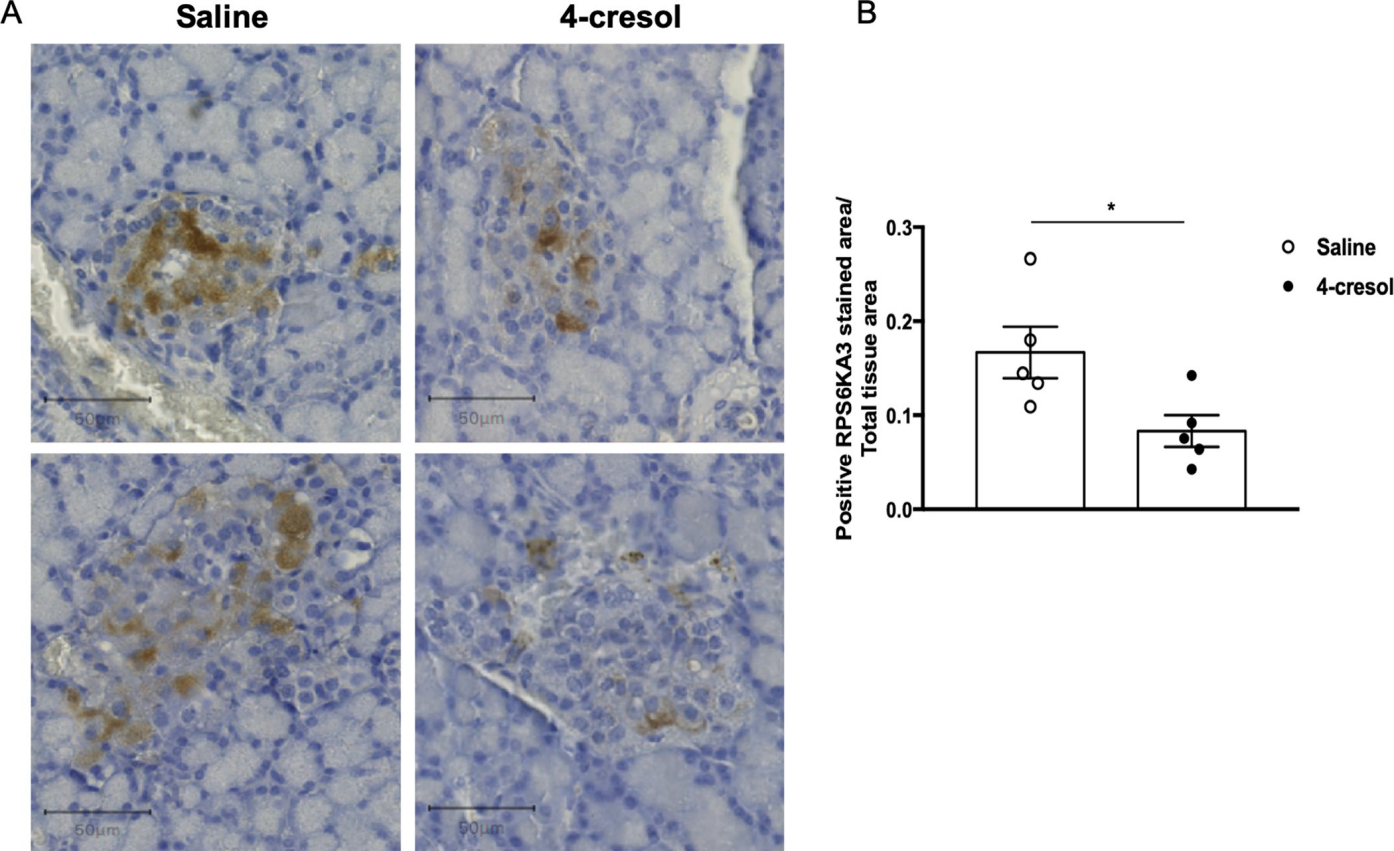
Chronic administration of 4-cresol in Goto-Kakizaki rats reduces RPS6KA3 expression in pancreatic islets. Spontaneously diabetic Goto-Kakizaki (GK) rats were chronically treated with subcutaneous infusion of 4-cresol (n = 6) or saline (n = 5) for 6-weeks. Pancreas sections were labeled with Hematoxylin-Eosin and immunohistochemistry using an antibody against RPS6KA3 to detect the protein (A) and determine its amount in islets (B). Results are expressed as the ratio of positive pixels corresponding to RPS6KA3 staining within islets and the total tissue area. Scale bars in (A) are 50 μm. Each dot in (B) represents the mean of three technical replicates per animal. Data were analyzed using the unpaired Mann-Whitney test. Results are means ± SEM. *P<0.05, significantly different between GK rats treated with 4-cresol or saline.

To investigate the presence of RPS6KA3 in rat pancreatic islets and test the possible impact of 4-cresol on its expression, we labelled the protein in pancreas sections of diabetic Goto-Kakizaki rats treated with either 4-cresol or saline [14]. As reported in humans, this kinase is highly expressed in rat pancreatic islets (**Fig 5A**). Chronic administration of 4-cresol *in vivo* resulted in a significant reduction in RPS6KA3 abundance in islets (**Fig 5B**), suggesting that the above reported *in vitro* interaction between 4-cresol and RPS6KA3 results in reduced expression of this kinase *in vivo*.

**Fig 6:**
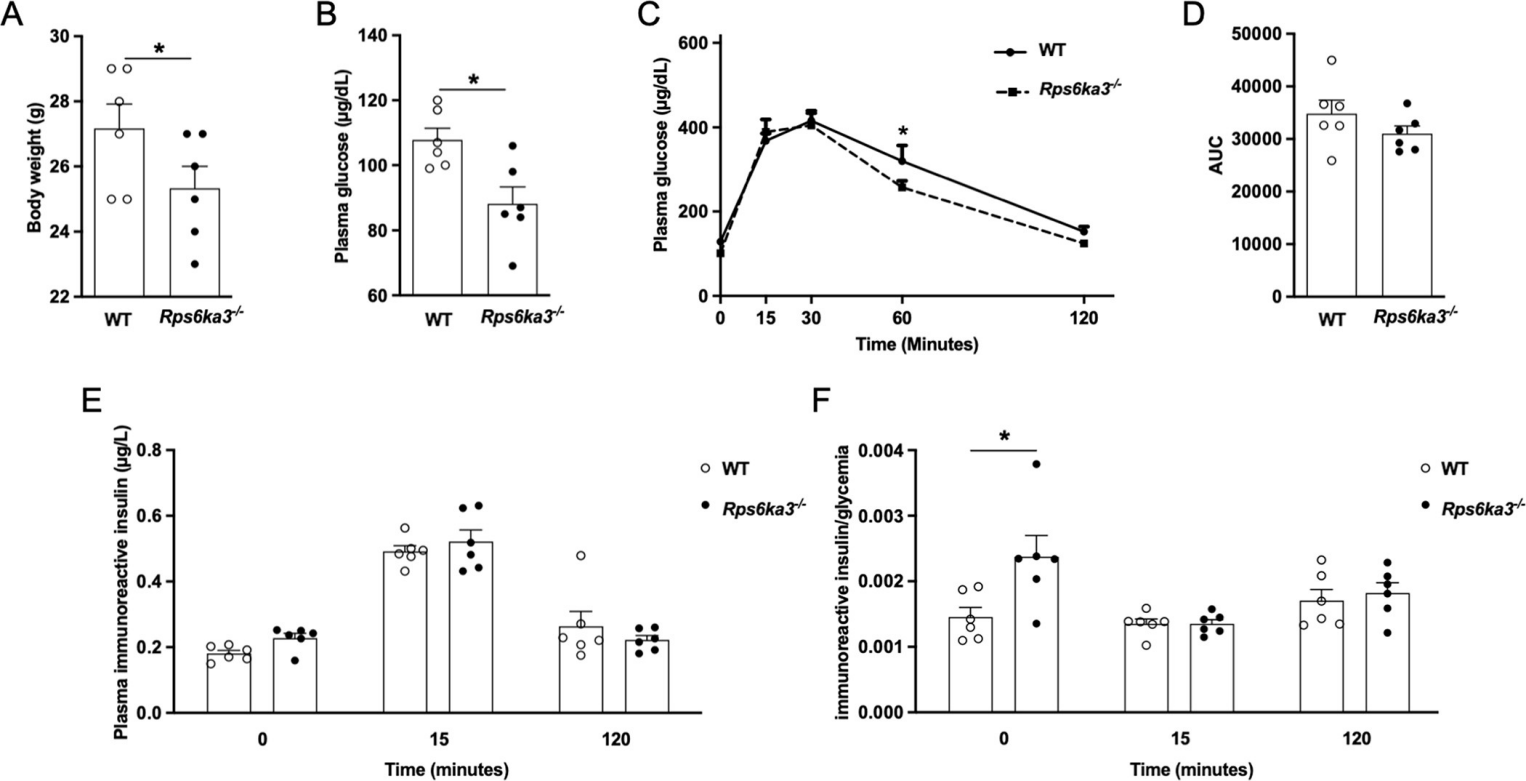
Body weight, glucose tolerance and insulin secretion in *Rps6ka3* deficient mice. Body weight (A), fasting glycemia (B), glucose tolerance (C,D) and insulin secretion (E,F) were determined in twenty week-old *Rps6ka3*^-/-^ (n = 6) and wild type (n = 6) mice. Intraperitoneal glucose tolerance tests (IPGTT) were performed to determine glycemic control (C,D) and glucose-stimulated insulin secretion expressed as plasma insulin values before glucose injection and 15 and 120 minutes afterwards (E) or ratios of insulin values to the corresponding values of plasma glucose during the IPGTT (F). Cumulative glycemia (Area Under the Curve, AUC) was calculated as the sum of plasma glucose values during the IPGTT (D). Data were analyzed using the unpaired Mann-Whitney test. Results are means ± SEM. *p<0.05 significantly different between *Rps6ka*3^**-/-**^ mice and controls.These results provide confirmatory evidence that RPS6KA3 is expressed in pancreatic islets. We demonstrate that *Rps6ka3* expression is reactive to *in vivo* prolonged exposure to 4-cresol and that *Rps6ka3* deficiency improves glycemic control and stimulates insulin production *in vivo*.

To test the hypothesis that inhibition of RPS6KA3 accounts for improved glucose homeostasis and enhanced insulin secretion mediated by 4-cresol, we carried out glucose tolerance and insulin secretion tests (IPGTT) in RPS6KA3 deficient mice. *Rps6ka3*^-/-^ mice showed a 9.2% reduction in body weight when compared to wild type controls (p = 0.05) (**Fig 6A**). Fasting blood glucose was significantly decreased in *Rps6ka3*^-/-^ mice when compared to controls (p = 0.02) (**Fig 6B**) and remained reduced prior to glucose injection. Glucose tolerance was improved in *Rps6ka3*^-/-^ mice as reflected by the significant reduction in glycemia 60 minutes after glucose injection in these mice when compared to controls (**Fig 6C**), which nevertheless resulted in a non significant reduction in the overall glucose levels during the IPGTT (**Fig 6D**). Insulin production prior to glucose stimulation and glucose induced insulin secretion were similar in the two mouse groups (**Fig 6E**). However, significantly higher insulin to glucose ratio prior to glucose injection in *Rps6ka3*^-/-^ mice than in controls suggests increased baseline insulin production (**Fig 6F**).

### RPS6KA3 inhibition alters lipid metabolism and β-cell function

**Fig 7:**
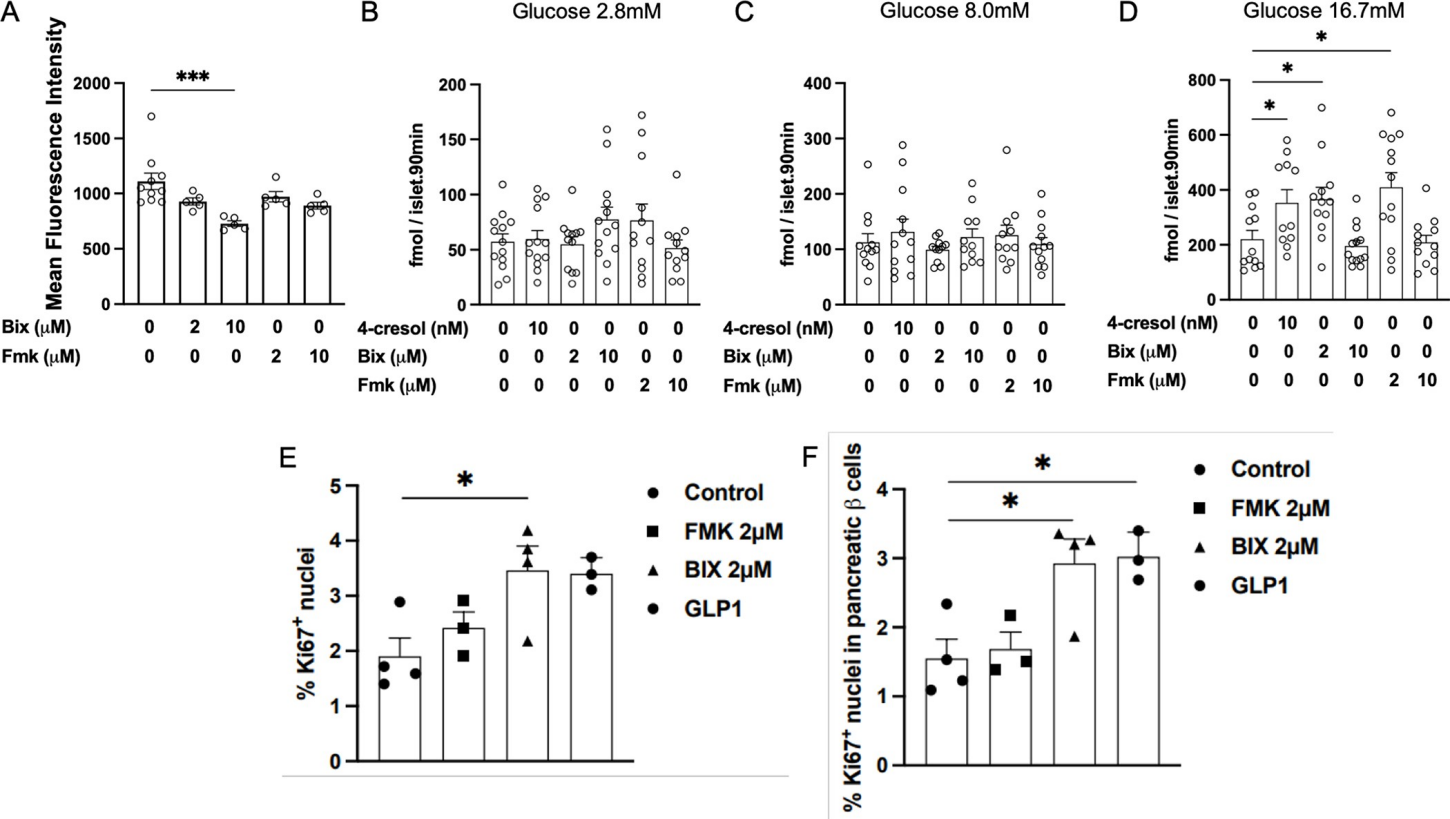
Effects of RPS6KA3 inhibitors on *in vitro* lipid metabolism and β–cell function. Lipid accumulation was determined on 3T3-L1 cells differentiated in adipocytes and incubated with BIX02565 or FMK (A). Insulin secretion was determined in rat isolated islets incubated with 4-cresol 10nM, BIX02565 (2 and 10 μM) or FMK (2 and 10μM) and glucose 2.8 mM (B), 8.0 mM (C) and 16.7 mM (D) glucose. Cellular proliferation was tested in rat isolated islets treated with BIX02565 (2 μM), FMK (2 μM) or GLP1 (100 nM) (E,F). Each point in (B-F) represents mean values of six islets. Results are means ± SEM. *p<0.05, ***p<0.001 significantly different to controls.These data provide information on a mechanism of action that may mediate the effects of 4-cresol on lipid metabolism and glucose homeostasis via inhibition of RPS6KA3. The use of inhibitors of this kinase mimics the action of 4-cresol on stimulated insulin secretion *in vitro* and provides important information on therapeutic pathways.

To provide further evidence of the role on RPS6KA3 in lipid metabolism and glucose homeostasis, we tested the impact of known inhibitors of this protein (BIX 02565 and FMK) on *in vitro* lipid metabolism in 3T3-L1 adipocytes and β-cell function in rat isolated islets. Incubation of 3T3-L1 cells with BIX 02565 2 μM and FMK 2 μM and 10 μM had no effects on lipid accumulation (**Fig 7A**). In contrast, treatment of the cells with BIX 02565 10 μM significantly reduces cellular lipids by 34.45% (728± 26 in 3T3-L1 treated cells and 1,111± 71 in control cells, p = 0.003) (**Fig 7A**).

Treatment of rat isolated islets with of 4-cresol at 10 nM and BIX 02565 and FMK at both 2 μM and 10 μM did not alter insulin production in response to 2.8 mM glucose (**Fig 7B**) and insulin secretion induced by glucose 8 mM (**Fig 7C**). In contrast 4-cresol at 10 nM significantly increased insulin secretion in response to glucose 16.7 mM by 60.37% when compared to control islets (p = 0.032) (**Fig 7D**). Similarly, insulin secretion induced by glucose 16.7 mM was significant increased in islets treated with BIX 02565 2 μM (+66.39%; 366 ± 44, p = 0.021) and FMK 2 μM (+85.98%; 410 ± 54, p = 0.013) when compared to controls (202 ± 32) (**Fig 7D**). Interestingly, higher concentrations of the RPS6KA3 inhibitors (10 μM) did not alter insulin secretion in response to 16.7 mM glucose when compared to controls (**Fig 7D**).

We then tested the effects of the RPS6KA3 inhibitors on β-cell proliferation at the concentration that resulted in increased insulin secretion. Incubation of islets with FMK had no effects on the proliferation of islet cells and β–cells (**Fig 7E and 7F**). In contrast, cell proliferation was significantly increased when islets were treated with BIX 02565 2 μM when compared to controls (**Fig 7E**). β–cell proliferation was significantly increased when compared to controls to a level similar of that of islets treated with GLP1 (**Fig 7F**).

## Discussion

We report results demonstrating the impact of low doses of 4-cresol on insulin sensitivity and stimulated insulin secretion induced by glucose *in vitro* and propose that downregulated islet expression and activity of the kinase RPS6KA3 contribute to its biological effects. These results from *in vitro* analyses complement knowledge of the impact of *in vivo* administration of 4-cresol on insulin sensitivity, improved glucose tolerance, stimulated insulin secretion, β-cell mass and β-cell proliferation, and reduced obesity and fatty liver that we demonstrated in preclinical models of type 2 diabetes [14].

Our data provide evidence of the lack of adverse effects of 4-cresol on insulin sensitivity and replicate its effects on enhanced insulin secretion when tested *in vitro* in mouse. Our previous findings of coincidental improvement of glucose tolerance and enhanced glucose induced insulin secretion *in vivo* in high fat diet fed mice and GK rats did not provide sufficient evidence of improved insulin sensitivity [14]. In physiological conditions of incubation in presence of insulin, low 4-cresol concentrations (10–30 nM) did not significantly alter insulin sensitivity in human primary adipocytes and potentiated insulin stimulated glucose uptake in C2C12 mouse myoblasts. Inhibition of insulin-stimulated glucose uptake was reported in mouse 3T3-L1 adipocytes incubated for 7 days in high concentrations of 4-cresol (100–200 μM) [19], and in mouse C2C12 myotubes with even higher levels of 4-cresol (370 nM) or p-cresyl sulfate (212 nM) [23,24]. Our results suggest that short time treatment with low concentrations of 4-cresol (10–30 nM) in presence of insulin improves insulin sensitivity in mouse myocytes.

We provide further evidence of the capacity of 4-cresol to strongly and significantly potentiate insulin response to glucose in mouse isolated islets. We were able to narrow down to 10 nM the concentration of 4-cresol able to potentiate glucose induced insulin secretion. Even though low concentration of 4-cresol (10 nM) was sufficient to induce this biological effect, as observed for insulin sensitivity, prolonged static incubation (48 hours) of islets with 4-cresol was required to significantly stimulate the secretion of insulin in response to glucose. This is consistent with our *in vivo* results in diabetic rats and mice, which showed that 4-cresol was able to significantly enhance insulin secretion when delivered chronically through osmotic minipumps [14]. Interestingly, incubation of mouse islets with 4-cresol at 10 nM induced a similar stimulation of insulin secretion induced by 16.7 mM glucose than GLP-1 at 100 nM.

Safety assessment of potential druggable targets is a crucial stage in the development of novel and efficient drug therapies [25]. The dose of 4-cresol chosen for *in vitro* pharmacological profiling is consistent with that associated with improved metabolic and hormonal parameters *in vivo* in mouse and rat models of type 2 diabetes (0.5 mg/kg/day) [14], which is well below doses known to induce toxicity. The lethal dose (LD50) for 4-cresol given orally ranges from 344 mg/kg/day in mice to 1460 mg/kg/day in rats [12]. The safety profiling of consensus targets that we applied to 4-cresol has the advantage of testing binding and functional impact with many molecular targets used by the pharmaceutical industry (G protein-coupled receptors, hormone receptors, ion channels, enzymes and transporters) in a single *in vitro* assay [26]. The wide variety of molecules included in the assay, which are associated with risk of many disease conditions and off-target drug effects, allowed us to predict absence of adverse effects of 4-cresol on multiple biological systems. These include lack of effects on targets important for cardiovascular and cerebrovascular diseases (eg. muscarinic receptors, 5-hydroxytryptamine receptor 1B), heart disease (eg. phosphodiesterase 3 inhibitors, 5-hydroxytryptamine receptor 2B), psychiatric and neurodegenerative disorders (eg. monoamine oxidase inhibitors, dopamine receptors), endocrine diseases (eg. androgen receptor), asthma and chronic obstructive pulmonary disease (eg. histamine H receptor), as well as those important for drug safety, including a lack of binding to the drug-induced long QT syndrome associated hERG1. Of note, 4-cresol did not show evidence of significant off target binding to adrenergic receptors α1A, α2A, β1 and β2, which regulate cardiac function and vascular tone and which bind BIX 02565, a potent RPS6KA3 inhibitor targeted for the treatment of heart failure [27].

Altered regulations of the signalling of protein kinases [28] and GPCRs [29] are central mechanisms involved in the etiopathogenesis of metabolic disorders. GPCRs can mediate cellular responses to specific metabolites, including immune responses and inflammation, and are targeted by many drugs. Kinases are also important therapeutic targets [30]. *In vitro* testing for interaction of 4-cresol to large panels of 241 GPCRs and 468 kinases provided information on candidate molecular mechanisms and signalling pathways that mediate its physiological effects. Several GPCRs that interact the most strongly with 4-cresol (>25% of protein activity), including SSTR5, GPR27 and GPR119, are expressed in β-cells and regulate insulin secretion [31–33], and may therefore at least partly contribute to its effects on β-cell function. The strongest evidence of inhibition of kinases by 4-cresol (91.2%) was obtained with the tyrosine kinase non receptor 1 (TNK1) (91.2%), the ribosomal protein S6 kinase A3 (RPS6KA3, RSK2) (66%) and the eukaryotic translation initiation factor 2 alpha kinase 4 (EIF2AK4, GCN2) (65%). The kinase DYRK1A, which we previously found to be significantly downregulated in pancreas of diabetic mice and GK rats chronically treated with 4-cresol and in isolated mouse islets exposed to 4-cresol [14], does not interact with 4-cresol (2% binding inhibition), indicating that 4-cresol affects DYRK1A signalling through inhibition of its transcription without altering its activity. TNK1 has been observed to promote apoptosis induced by the tumor necrosis factor alpha [34] and its activity has been shown to stimulate growth and survival of pancreatic cancer cells [35]. EIF2AK4 is activated upon amino acid deficiency, resulting in downregulation of protein translation through phosphorylation of the eukaryotic translation initiation factor-2α, activation of the transcription factor ATF4 and up-regulation of autophagy [36]. RPS6KA3 also phosphorylates ATF4 [21] and is involved in the MAPK pathway through binding to ERK1/2, which regulates β-cell function [37], and in osteoblast differentiation through mitogen-induced c-Fos transcription regulation [38].

All three kinases binding 4-cresol show low tissue specificity in humans (www.proteinatlas.org). However, RPS6KA3 is strongly expressed in human pancreatic islets (www.proteinatlas.org/ENSG00000177189-RPS6KA3/tissue/pancreas), which we verified in rodents, and is therefore a prime candidate to account for the impact of 4-cresol on the regulation of β-cell function. Its high expression in exocrine pancreas may account for increased pancreas weight in diabetic rats and mice chronically treated with 4-cresol [14]. We found that RPS6KA3 is downregulated in islets from GK rats treated with 4-cresol and that *Rps6ka3* deficiency in mice is associated with modest, though significant, improvement of glucose tolerance and elevated insulin production. In addition, we show that both RPS6KA3 inhibitors FMK and BIX 02565 stimulate *in vitro* glucose stimulated insulin secretion. Interestingly, *Rps6ka3* knockdown in the α-cell line αTC1 results in insulin gene expression [39]. Previous studies showed that *Rps6ka3*^-/-^ mice exhibit increased insulin-stimulated glycogen synthase activity in muscle, elevated fasting insulin levels and a 10% reduction of body weight to a similar level than in our study (9.2%) [40,41]. However, these mice exhibited marked glucose intolerance [40], which is in sharp contrast with our results and could be due to genetic differences in the two knock out models. Moreover, the observation that the RPS6KA3 inhibitor BIX 02565 increases β-cell proliferation aligns with our previous results showing that 4-cresol similarly increases β-cell proliferation [14], consistent with the hypothesis that 4-cresol mediates certain effects via reduced RPS6KA3 function. The specific effect of BIX 02565 on β-cell proliferation may be explained by differences in the spectrum of proteins targeted by the two RPS6KA3 inhibitors.

In conclusion, our findings further document the effects of low doses of 4-cresol on enhanced insulin secretion and action when tested *in vitro* and uncover RPS6KA3 as a potential molecular target mediating its physiological roles in β-cell function. These results support the exploitation of 4-cresol in therapeutic applications in syndromes of insulin deficiency.

### Limitations of the study

The modest magnitude of changes in glucose homeostasis in our *Rps6ka3*^-/-^ mice suggests the involvement of other protein mediators in the function of 4-cresol in islets, which requires further molecular investigations. In addition, considering the widespread expression of RPS6KA3, TNK1 and EIF2AK4 in many tissues, exposure to 4-cresol may trigger a broad spectrum of biological responses, which warrant multi-organ physiological and molecular analyses. It may be relevant to multiple associations of one of its products (4-cresyl sulfate) with a broad range of phenotypes in humans [17]. Interaction of 4-cresol with these proteins, as well as with as yet unknown mediators, in other organs than islets may account for its pleiotropic effects on improved glycemic control and reduced adiposity and fatty liver in high fat diet fed mice [14]. Of note, mice deficient in EIF2AK4 exhibit resistance to obesity and hepatic steatosis induced by high fat diet [42] and streptozotocin-induced diabetes is associated with elevated abundance and activity of RPS6KA3 in skeletal muscle [43].

## Supporting information

S1 Table Summary of agonist and antagonist activities for 4-cresol tested in the gpcrMAX and orphanMax panels of known and orphan G protein-coupled receptors (GPCRs), respectively. The effects were tested at at concentration of 10μM of 4-cresol. The known GPCRs were tested in both agonist and antagonist assay modes, whereas the orphan GPCRs were tested antagonist assay moce. The measured effects (%) correpond to the percentage of remaining activity for the agonist assay and the percentage of inhibition for the antagonist assay.
(XLSX)

S2 Table Details of the 468 kinases tested in the KINOMEscan interaction assays and percentage of binding inhibition to the proteins by 4-cresol (10μM). (XLSX)

## References

[1] Buniello A. et al., The NHGRI-EBI GWAS Catalog of published genome-wide association studies, targeted arrays and summary statistics 2019. *Nucleic Acids Res* 47, D1005–D1012 (2019). doi: 10.1093/nar/gky1120 30445434 PMC6323933

[2] Visscher P. M. et al., 10 Years of GWAS Discovery: Biology, Function, and Translation. *Am J Hum Genet* 101, 5–22 (2017). doi: 10.1016/j.ajhg.2017.06.005 28686856 PMC5501872

[3] Srour B. et al., Ultra-processed food intake and risk of cardiovascular disease: prospective cohort study (NutriNet-Santé). *BMJ* 365, l1451 (2019). 31142457 10.1136/bmj.l1451 PMC6538975

[4] Fiolet T. et al., Consumption of ultra-processed foods and cancer risk: results from NutriNet-Santé prospective cohort. *BMJ* 360, k322 (2018). 29444771 10.1136/bmj.k322 PMC5811844

[5] Suez J. et al., Artificial sweeteners induce glucose intolerance by altering the gut microbiota. *Nature* 514, 181–186 (2014). doi: 10.1038/nature13793 25231862

[6] Chassaing B. et al., Dietary emulsifiers impact the mouse gut microbiota promoting colitis and metabolic syndrome. *Nature* 519, 92–96 (2015). doi: 10.1038/nature14232 25731162 PMC4910713

[7] Sargis R. M., Simmons R. A., Environmental neglect: endocrine disruptors as underappreciated but potentially modifiable diabetes risk factors. *Diabetologia* 62, 1811–1822 (2019). doi: 10.1007/s00125-019-4940-z 31451869 PMC7462102

[8] Barouki R., Audouze K., Coumoul X., Demenais F., Gauguier D., Integration of the human exposome with the human genome to advance medicine. *Biochimie* 152, 155–158 (2018). doi: 10.1016/j.biochi.2018.06.023 29960033

[9] Nicholson J. K. et al., Host-gut microbiota metabolic interactions. *Science* 336, 1262–1267 (2012). doi: 10.1126/science.1223813 22674330

[10] Passmore I. J. et al., Para-cresol production by Clostridium difficile affects microbial diversity and membrane integrity of Gram-negative bacteria. *PLoS Pathog* 14, e1007191 (2018). doi: 10.1371/journal.ppat.1007191 30208103 PMC6135563

[11] Dawson L. F. et al., The analysis of para-cresol production and tolerance in Clostridium difficile 027 and 012 strains. *BMC Microbiol* 11, 86 (2011). doi: 10.1186/1471-2180-11-86 21527013 PMC3102038

[12] Andersen A., Final report on the safety assessment of sodium p-chloro-m-cresol, p-chloro-m-cresol, chlorothymol, mixed cresols, m-cresol, o-cresol, p-cresol, isopropyl cresols, thymol, o-cymen-5-ol, and carvacrol. *Int J Toxicol* 25 Suppl 1, 29–127 (2006). doi: 10.1080/10915810600716653 16835130

[13] Dietz D., NTP technical report on the toxicity studies of Cresols (CAS Nos. 95-48-7, 108-39-4, 106-44-5) in F344/N Rats and B6C3F1 Mice (Feed Studies). *Toxic Rep Ser* 9, 1–128 (1991). 12209177

[14] Brial F. et al., The Natural Metabolite 4-Cresol Improves Glucose Homeostasis and Enhances β-Cell Function. *Cell Rep* 30, 2306–2320.e2305 (2020). 32075738 10.1016/j.celrep.2020.01.066

[15] Bermudez-Martin P. et al., The microbial metabolite p-Cresol induces autistic-like behaviors in mice by remodeling the gut microbiota. *Microbiome* 9, 157 (2021). doi: 10.1186/s40168-021-01103-z 34238386 PMC8268286

[16] Toft P. B. et al., Microbial metabolite p-cresol inhibits gut hormone expression and regulates small intestinal transit in mice. *Front Endocrinol (Lausanne)* 14, 1200391 (2023). doi: 10.3389/fendo.2023.1200391 37534214 PMC10391832

[17] Ou H. et al., A phenome-wide association study (PheWAS) to identify the health impacts of 4-cresol sulfate in the Nagahama Study. *Sci Rep* 13, 13926 (2023). doi: 10.1038/s41598-023-40697-2 37626071 PMC10457396

[18] Elliott P. et al., Urinary metabolic signatures of human adiposity. *Sci Transl Med* 7, 285ra262 (2015). doi: 10.1126/scitranslmed.aaa5680 25925681 PMC6598200

[19] Tanaka S., Yano S., Sheikh A. M., Nagai A., Sugimoto T., Effects of uremic toxin p-cresol on proliferation, apoptosis, differentiation, and glucose uptake in 3T3-L1 cells. *Artif Organs* 38, 566–571 (2014). doi: 10.1111/aor.12252 24417700

[20] Bartlett D. E. et al., Uremic Toxins Activates Na/K-ATPase Oxidant Amplification Loop Causing Phenotypic Changes in Adipocytes in In Vitro Models. *Int J Mol Sci* 19, (2018). doi: 10.3390/ijms19092685 30201874 PMC6164729

[21] Yang X. et al., ATF4 is a substrate of RSK2 and an essential regulator of osteoblast biology; implication for Coffin-Lowry Syndrome. *Cell* 117, 387–398 (2004). doi: 10.1016/s0092-8674(04)00344-7 15109498

[22] Han J. H., Kim S., Lee H., Park S. Y., Woo C. H., FMK, an Inhibitor of p90RSK, Inhibits High Glucose-Induced TXNIP Expression via Regulation of ChREBP in Pancreatic β Cells. *Int J Mol Sci* 20, (2019). 10.3390/ijms20184424 PMC6770409 31505737

[23] Koppe L. et al., p-Cresyl sulfate promotes insulin resistance associated with CKD. *J Am Soc Nephrol* 24, 88–99 (2013). doi: 10.1681/ASN.2012050503 23274953 PMC3537215

[24] Koppe L. et al., p-Cresyl glucuronide is a major metabolite of p-cresol in mouse: in contrast to p-cresyl sulphate, p-cresyl glucuronide fails to promote insulin resistance. *Nephrol Dial Transplant* 32, 2000–2009 (2017). doi: 10.1093/ndt/gfx089 28992089

[25] Bass A. S. et al., Exploratory drug safety: a discovery strategy to reduce attrition in development. *J Pharmacol Toxicol Methods* 60, 69–78 (2009). doi: 10.1016/j.vascn.2009.04.194 19422924

[26] Bowes J. et al., Reducing safety-related drug attrition: the use of in vitro pharmacological profiling. *Nat Rev Drug Discov* 11, 909–922 (2012). doi: 10.1038/nrd3845 23197038

[27] Fryer R. M. et al., Mitigation of off-target adrenergic binding and effects on cardiovascular function in the discovery of novel ribosomal S6 kinase 2 inhibitors. *J Pharmacol Exp Ther* 340, 492–500 (2012). doi: 10.1124/jpet.111.189365 22128344

[28] Lahiry P., Torkamani A., Schork N. J., Hegele R. A., Kinase mutations in human disease: interpreting genotype-phenotype relationships. *Nat Rev Genet* 11, 60–74 (2010). doi: 10.1038/nrg2707 20019687

[29] Ghislain J., Poitout V., Targeting lipid GPCRs to treat type 2 diabetes mellitus—progress and challenges. *Nat Rev Endocrinol* 17, 162–175 (2021). 33495605 10.1038/s41574-020-00459-w

[30] Klaeger S. et al., The target landscape of clinical kinase drugs. *Science* 358, (2017). doi: 10.1126/science.aan4368 29191878 PMC6542668

[31] Ku G. M., Pappalardo Z., Luo C. C., German M. S., McManus M. T., An siRNA screen in pancreatic beta cells reveals a role for Gpr27 in insulin production. *PLoS Genet* 8, e1002449 (2012). doi: 10.1371/journal.pgen.1002449 22253604 PMC3257298

[32] Ludvigsen E., Olsson R., Stridsberg M., Janson E. T., Sandler S., Expression and distribution of somatostatin receptor subtypes in the pancreatic islets of mice and rats. *J Histochem Cytochem* 52, 391–400 (2004). doi: 10.1177/002215540405200310 14966206

[33] Panaro B. L. et al., β-Cell Inactivation of. *Diabetes* 66, 1626–1635 (2017). 28254842 10.2337/db17-0017 PMC5860191

[34] Azoitei N. et al., Thirty-eight-negative kinase 1 (TNK1) facilitates TNFalpha-induced apoptosis by blocking NF-kappaB activation. *Oncogene* 26, 6536–6545 (2007). doi: 10.1038/sj.onc.1210476 17471239

[35] Henderson M. C. et al., High-throughput RNAi screening identifies a role for TNK1 in growth and survival of pancreatic cancer cells. *Mol Cancer Res* 9, 724–732 (2011). doi: 10.1158/1541-7786.MCR-10-0436 21536687 PMC3137903

[36] Masson G. R., Towards a model of GCN2 activation. *Biochem Soc Trans* 47, 1481–1488 (2019). doi: 10.1042/BST20190331 31647517 PMC6824675

[37] Lawrence M., Shao C., Duan L., McGlynn K., Cobb M. H., The protein kinases ERK1/2 and their roles in pancreatic beta cells. *Acta Physiol (Oxf)* 192, 11–17 (2008). doi: 10.1111/j.1748-1716.2007.01785.x 18171425

[38] Romeo Y., Zhang X., Roux P. P., Regulation and function of the RSK family of protein kinases. *Biochem J* 441, 553–569 (2012). doi: 10.1042/BJ20110289 22187936

[39] Fomina-Yadlin D. et al., Small-molecule inducers of insulin expression in pancreatic alpha-cells. *Proc Natl Acad Sci U S A* 107, 15099–15104 (2010). doi: 10.1073/pnas.1010018107 20696901 PMC2930573

[40] El-Haschimi K. et al., Insulin resistance and lipodystrophy in mice lacking ribosomal S6 kinase 2. *Diabetes* 52, 1340–1346 (2003). doi: 10.2337/diabetes.52.6.1340 12765942

[41] Dufresne S. D. et al., Altered extracellular signal-regulated kinase signaling and glycogen metabolism in skeletal muscle from p90 ribosomal S6 kinase 2 knockout mice. *Mol Cell Biol* 21, 81–87 (2001). doi: 10.1128/MCB.21.1.81-87.2001 11113183 PMC88782

[42] Liu S. et al., GCN2 deficiency protects against high fat diet induced hepatic steatosis and insulin resistance in mice. *Biochim Biophys Acta Mol Basis Dis* 1864, 3257–3267 (2018). doi: 10.1016/j.bbadis.2018.07.012 30006154

[43] Markuns J. F. et al., Effects of streptozocin-induced diabetes and islet cell transplantation on insulin signaling in rat skeletal muscle. *Endocrinology* 140, 106–111 (1999). doi: 10.1210/endo.140.1.6427 9886813

